# IMPACT OF FLUORESCENT DYES ON MUTATIONS IN NEXT GENERATION SEQUENCING LIBRARY GENERATION

**DOI:** 10.7171/001c.166072

**Published:** 2026-09-01

**Authors:** Vincent Butty, Pranav Patel, Stefan Green, Stuart Levine

**Affiliations:** 1 Biology Massachusetts Institute of Technology; 2 Biological Engineering Massachusetts Institute of Technology; 3 Koch Institute for Integrative Cancer Research Massachusetts Institute of Technology; 4 NA n6TEC; 5 Genomics and Microbiome Core Facility Rush University Medical Center

**Keywords:** qPCR, NGS, Library Quantification, Mutation Signature, 16S

## Abstract

DNA labelling fluorescent dyes such as ethidium bromide have long been considered to be highly mutagenic during DNA replication. While recent studies have pushed back on this narrative, the intercalative nature of these dyes continues to raise the possibility that these dyes can induce mutations. The iconPCR instrument by n6tec uses fluorescent dyes to measure amplification in real time and to adjust cycling conditions. However, since this use of qPCR is preparative and not analytical, mutations introduced by fluorescent dyes would be propagated into the sequencing reaction. To address the impact of these dyes on downstream analyses, we have performed routine mutation calling as well as mutational signature analysis on samples amplified using the iconPCR in the presence of either SYBR or EvaGreen. Sequence analysis revealed very minimal impacts of dyes on the reactions, largely within the noise regimen with only subtle changes in mutation rates seen. Mutational signature analysis was unable to identify any key signatures assignable to the dyes in either substitutions or indel domains. The mutational impact of intercalating dyes during fluorescence-guided amplification is therefore minimal and can be disregarded in all but the most sensitive NGS applications.

## Introduction

Intercalating dyes have been used to detect and measure DNA for several decades.^1^ These dyes fluoresce robustly in the context of double-stranded DNA, and the variable fluorescence led to the invention of quantitative PCR, which incorporates optical detection into the typical PCR reaction to measure the amount of fluorescence after each cycle. By measuring the cycle at which fluorescence crosses a set threshold, these instruments quantify the amount of input material included in each reaction. The intercalating mechanism of these dyes, inserting between adjacent base pairs and distorting the DNA helix, raises a direct concern regarding mutagenicity. Intercalating dyes have long been classified as suspected mutagens and possible carcinogens in laboratory safety documentation, a classification supported by early bacterial mutagenicity assays.^2^ More recent studies have found that mutagenic potential requires metabolic activation and may be substantially lower than initially feared,^3^ though the possibility that these dyes could introduce errors during DNA replication remains an open question.

The n6tec iconPCR (Individually Controlled PCR) instrument (n6tec, Pleasanton, CA, USA) takes quantitative PCR applications a step further. Rather than applying uniform cycling conditions across an entire plate, the instrument independently monitors and controls each of 96 wells in real time, halting individual reactions as they exit the exponential amplification phase or meet other user-specified conditions. This per-well control offers a significant advantage over conventional fixed-cycle thermocyclers in next-generation sequencing (NGS) library preparation, where both over- and underamplification of libraries presents critical challenges. Underamplification yields insufficient material and may necessitate re-amplification, introducing both delay and variability. Over-amplification, particularly reactions driven into the PCR plateau phase, is associated with GC and composition bias, increased duplicate rates, chimera formation, and the generation of spurious higher-molecular-weight heteroduplex products, all of which can compromise library pooling and downstream sequencing analyses.^4–6^

The use of intercalating dyes in the iconPCR instrument (n6tec) therefore raises a specific concern: dye-induced errors introduced during preparative amplification would be propagated into the sequencing library and could confound downstream analyses. To assess this risk, we employed mutational signature analysis alongside conventional variant calling in a simple experimental system. Mutational signatures are characteristic combinations of mutation types arising from specific mutagenic processes; a mutagen acting through a defined mechanism, such as base intercalation, would be expected to produce a recognizable pattern of mutations attributable to that mechanism.^7^ We therefore performed detailed analysis of reference samples prepared using the iconPCR instrument (n6tec) in the presence of intercalating dyes and compared to controls amplified without dyes. Bulk analysis revealed minimal changes in both single-nucleotide variation rates and insertion or deletion frequencies. Mutational signature analysis revealed minimal signatures not associated with known mechanisms. Thus, amplification with the iconPCR instrument (n6tec) should have minimal impact on next-generation sequencing (NGS) analyses except in the most sensitive applications.

## Materials and Methods

### Human Genomic DNA and Library Preparation

Human genomic DNA from Genome In A Bottle (GIAB) reference material NA12878 was obtained from the Coriell Institute (Camden, NJ, USA).^8,9^ DNA was enzymatically fragmented to a target insert size of approximately 150–200 bp using Ultra II FS DNA Library Prep kit (New England Biolabs, Ipswich, MA, USA, catalog no. E7805). Following fragmentation, end repair, dA-tailing, and ligation of P5/P7 Illumina-compatible adapters (New England Biolabs) were performed per the manufacturer’s protocol. Ligation products were purified using NEBNext Sample Purification Beads (New England Biolabs, catalog no. E6552A), at a 0.8X ratio.

### IconPCR Amplification

Amplification was performed on an iconPCR instrument (n6tec) using NEB Q5 High-Fidelity DNA Polymerase (New England Biolabs). 2.5ng of ligated material was amplified in the presence of SYBR Green I (Thermo Fisher Scientific), EvaGreen (Biotium), or EvaGreen Plus (Biotium) at 1X (final concentration) alongside a no-dye control, for 10 cycles under the following conditions: initial denaturation at 98°C for 30 seconds, 10 cycles at 98°C for 10 seconds and 65°C for 75 seconds, final extension at 65°C for 5 minutes and hold at 4°C. Dye concentrations and cycle counts were intentionally set above the iconPCR instrument’s (n6tec) standard stop point parameters to provide a stringent test of mutagenic potential. Amplified libraries were purified using NEBNext Sample Purification Beads (New England Biolabs), at a 0.8X ratio.

### Sequencing, Alignment, and Quality Control

Libraries were quantified using an Agilent Fragment Analyzer (Agilent Technologies, Santa Clara, CA, USA) and by qPCR and sequenced on an Element AVITI using 2 x 75 nt reads to a depth of approximately 100 million reads per sample (93–138M). Sequencing reads from the four conditions were aligned against GRCh38 using BWA-MEM (VN:0.7.17-r1188).^10^ Quality control metrics were calculated (including number of aligned reads, multiply-mapping reads, and number of unique 20-mers in the top 10 million reads) and samples were checked for contamination against a collection of reference genomes.

### Variant Calling

Each sample was mapped against the GIAB GRCh38-based reference assembly GRCh38_GIABv3_no_alt_analysis_set_maskedGRC_decoys_MAP2K3_KMT2C_KCNJ18.fasta, obtained from https://ftp-trace.ncbi.nlm.nih.gov/giab/ftp/release/references/, using BWA-MEM (v. 0.7.12-r1039, -t 16 cores).^10^ Resulting BAM files were sorted and indexed using SAMtools v.1.3. Variant calling was performed using BCFtools mpileup (v. 1.10.2+htslib-1.10.2) with parameters -B -x -d 1000000 –threads 8 -A -O v, against the above reference assembly.^11,12^ VCF files were filtered against the GIAB benchmark VCF for sample NA12878_HG001 (HG001_GRCh38_1_22_v4.2.1_benchmark.vcf.gz, retrieved from https://ftp-trace.ncbi.nlm.nih.gov/ReferenceSamples/giab/release/NA12878_HG001/latest/GRCh38/) using the BCFtools isec function (flags -c all -O v).^9^ No additional filtering was performed. SNV rates were calculated as total observed variant bases divided by total bases sequenced; indel rates were calculated analogously.

### 16S rRNA Gene Amplicon Library Preparation

A 16S rRNA gene amplicon control was generated from the ZymoBIOMICS Microbial Community DNA Standard (Zymo Research, Irvine, CA, USA; catalog no. D6306) using a two-stage PCR protocol as described previously.^13^ The V4–V5 region of the microbial small subunit rRNA gene was targeted using primers sIDTP5_515F and sIDTP7_926R (CTACACGACGCTCTTCCGATCTGTGYCAGCMGCCGCGGTAA and CAGACGTGTGCTCTTCCGATCTCCGYCAATTYMTTTRAGTTT, respectively; underlined regions represent linker sequences), which carry 5′ common sequence tags (sIDTP5 and sIDTP7) matching 3′ sequences present in IDT xGen Amplicon UDI primers (Integrated DNA Technologies, Coralville, IA, USA). First-stage amplifications were performed in 10 µL reactions using repliQa HiFi ToughMix (Quantabio, Beverly, MA, USA), with template added at 5 ng/reaction, in the presence or absence of SYBR Green I Nucleic Acid Gel Stain (Thermo Fisher Scientific; catalog no. S7563) at 1X working concentration. Cycling conditions were 98°C for two minutes, followed by 16, 20, 24, or 28 cycles of 98°C for 10 seconds, 50°C for one second, and 68°C for one second. A second-stage amplification was then performed in 10 µL reactions using repliQa HiFi ToughMix (Quantabio), with each well receiving a unique dual-index primer pair (IDT xGen Amplicon UDI primer sets). One µL of first-stage product was used as template without cleanup, with 2 µL of primer per reaction. Cycling conditions were 98°C for two minutes, followed by eight cycles of 98°C for 10 seconds, 60°C for one second, and 68°C for 1 second. PCR products were pooled in equal volumes, purified using a 0.6X AMPure ratio, and sequenced with a 10% PhiX spike-in on an Illumina MiSeq i100+ instrument (Illumina, San Diego, CA, USA) using a 600-cycle kit with read lengths extended to 2 x 309 bases.

### *16S* Error Rate Analysis

Reads were mapped against the reference *16S* and *18S rRNA* gene sequences provided for the ZymoBIOMICS Microbial Community DNA Standard, after removing duplicate gene copies and masking positions at which distinct copies of the same species differed; masked positions were excluded from both the numerator and denominator of all error rate calculations. Paired-end reads were merged using BBMerge,^14^ exploiting the overlap of the V4–V5 insert to suppress independent sequencer errors by consensus; read pairs without a confident overlap were discarded. Merged reads were trimmed to remove primer sequences using Cutadapt^15^ with the –discard-untrimmed flag, and reads shorter than 100 bp after trimming were discarded. Trimmed reads were aligned to the deduplicated reference using BWA-MEM^10^ and coordinate-sorted using SAMtools^12^; unmapped, secondary, and supplementary alignments were excluded. Per-base error counts were extracted from sorted, indexed BAM files using a custom Python script (pysam^12^). Substitution events were classified in the canonical pyrimidine context by strand collapse, enumerating six substitution classes (C>A, C>G, C>T, T>A, T>C, T>G); C-context rates were computed as events divided by total covered C and G positions (excluding masked positions), and T-context rates analogously over T and A positions. Insertion and deletion rates were computed as the number of inserted or deleted bases divided by the total number of covered reference positions, excluding masked positions.

### Mutational Signature Analysis

Variant positions, reference and derived alleles from condition-specific VCF files were processed using the Signal mutational spectra elicitation webservice for single and double substitutions and indels (https://signal.mutationalsignatures.com/).^16,17^ Signature reconstruction was first performed on the absolute mutational spectra of each condition using SigProfilerAssignment (COSMIC v3.4, default activity thresholds).^18^ Dye-imparted mutational spectra were then assessed by subtracting the frequency of each variant class in the no-dye control from the corresponding frequencies in each of the three dye-treated samples. These difference frequencies were multiplied by the number of observed sequence variants of the corresponding class in each dye-treated sample, rounded, and floored to zero, then submitted as 96-, 78-, or 89-channel variation catalogs to Signal. Signature reconstruction was repeated on these different spectra using SigProfilerAssignment with the same parameters. Indel classes were assessed using a recently published taxonomy.^19^ For base count level processing, non-reference sites were annotated using the Signal’s companion library signature.tools.lib (https://github.com/Nik-Zainal-Group/signature.tools.lib), including the indelsig.tools.lib-contained functions in the R statistical environment (v. 4.2.0). Corresponding reference and derived allele counts were retrieved from the original VCF files and tallied as the above variation catalogs for downstream processing in Signal.

## Results

**Figure 1.**
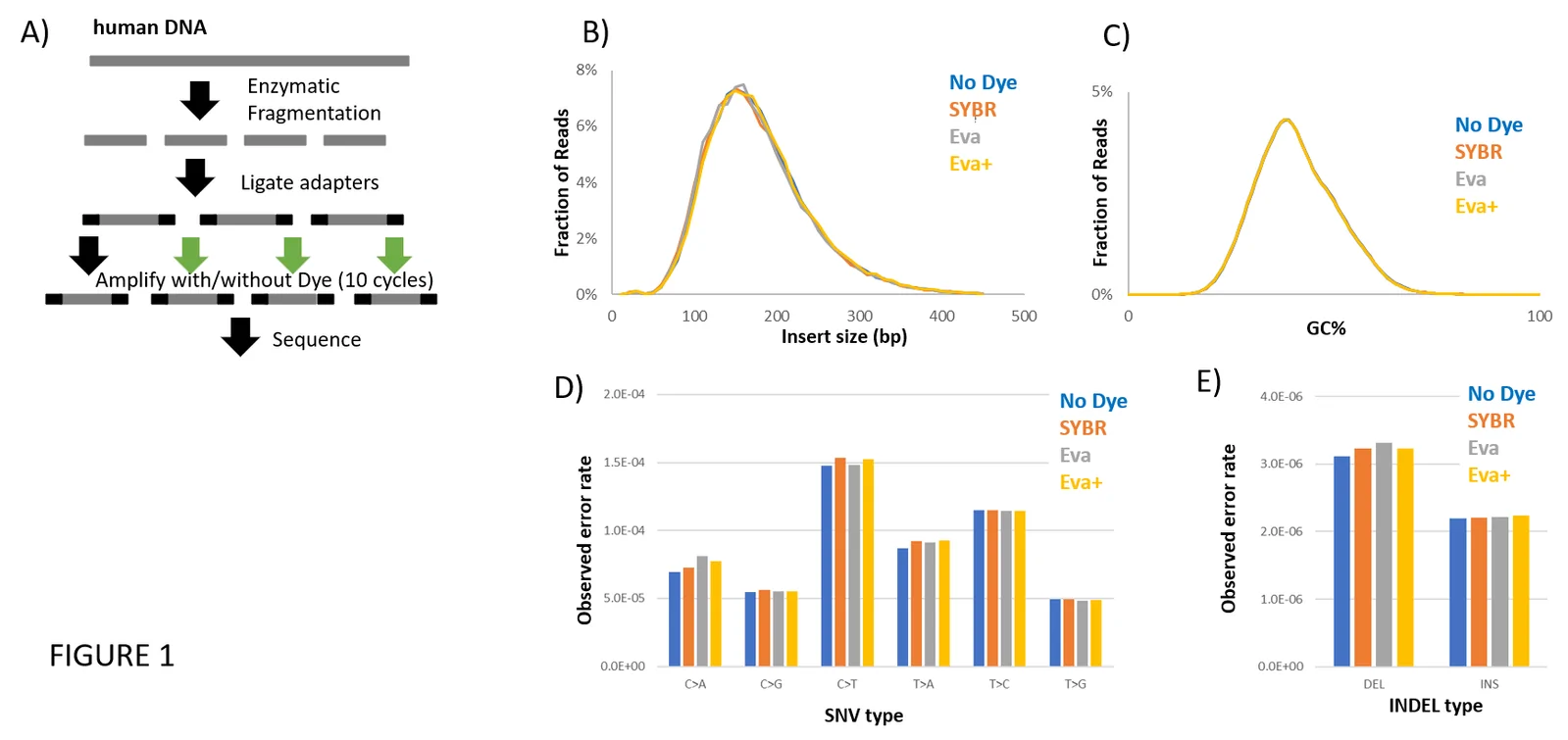
**Impact of intercalating dyes on NGS library quality and mutation rates.** (A) Schematic of the experimental workflow. (B) Histogram of insert lengths (bp) for libraries prepared under each dye condition. SYBR: SYBR Green I; Eva: EvaGreen; Eva+: EvaGreen Plus. (C) Frequency distribution of GC fraction for libraries prepared under each dye condition. (D) Observed single-base mutational error rates by substitution type (C>A, C>G, C>T, T>A, T>C, T>G), normalized to total bases sequenced, for libraries prepared under each dye condition. (E) Observed indel error rates by event type (deletion, insertion), normalized to total bases sequenced, for libraries prepared under each dye condition.

To evaluate the gross impact of intercalating dyes present in the amplification reaction on NGS library quality, P5/P7-anchored libraries were prepared from enzymatically sheared human genomic DNA (GIAB reference sample NA12878) in the presence of SYBR Green I (Thermo Fisher Scientific), EvaGreen (Biotium), or EvaGreen Plus (Biotium) using NEB Q5 High-Fidelity DNA Polymerase on an iconPCR instrument (n6tec) (Figure 1A). Relatively high concentrations of dye and cycle counts that exceed the standard stop point of the iconPCR instrument (n6tec) were selected to amplify any mutagenicity from the dyes while remaining within its performance envelope. Libraries were sequenced on an Element AVITI (Element Biosciences) to maximize sensitivity to any dye-induced mutations. Global library metrics showed that dye-containing reactions produced libraries of comparable quality to the no-dye control, with only subtle differences in insert size distribution (Figure 1B) and GC content (Figure 1C) observed.

The primary impact of intercalating dyes was expected to be in the induction of mutations. Single-base mutational rates were characterized relative to the total number of bases sequenced (since all errors are expected to be technical in origin). Mutation analysis found very similar error rates among all the samples, regardless of the presence of dyes, with dye-containing samples increasing the error rate by less than 3.5% above the no-dye control, with SYBR Green I (Thermo Fisher Scientific) showing slightly lower substitution error rates relative to EvaGreen (Biotium) formulations. Base-by-base substitution errors showed similar rates of transitions and transversions (Figure 1D). Indel mutation rates were also only slightly perturbed by the presence of the dyes with ~5% more deletions and 1–2% additional insertions observed, but still extremely close to background rates (Figure 1E).

**Figure 2.**
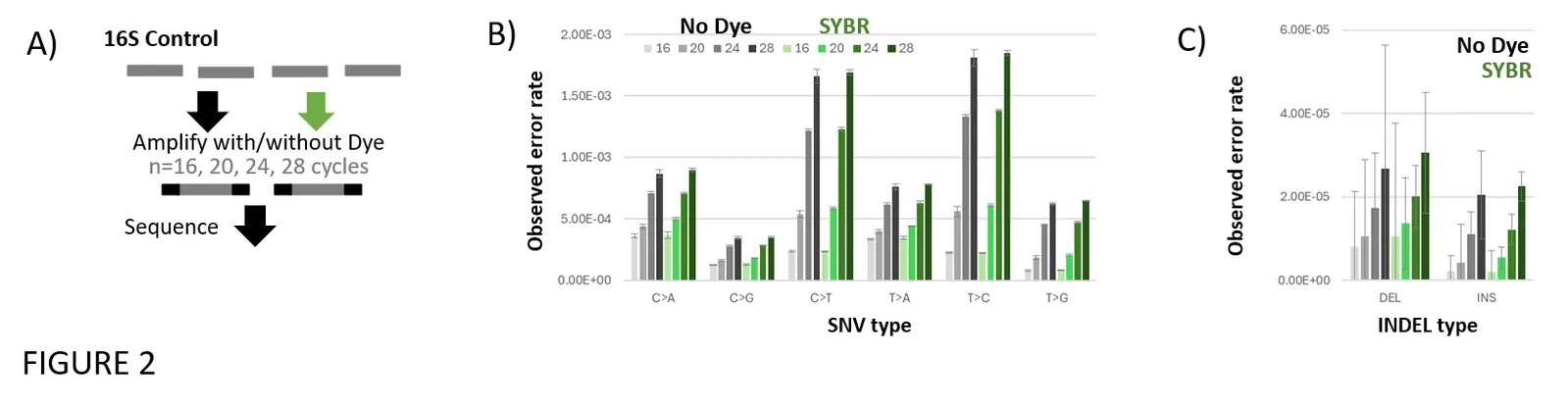
**Impact of cycle number and intercalating dye on mutation rates in 16S amplicon sequencing.** (A) Schematic of the experimental workflow. (B) Observed single-base mutational error rates by substitution type (C>A, C>G, C>T, T>A, T>C, T>G), normalized to total bases sequenced, for each cycle number (16, 20, 24, 28) with and without SYBR Green I. No dye: gray; SYBR Green I: green. (C) Observed indel error rates by event type (deletion, insertion), normalized to total bases sequenced, for each condition as in (B).

Because many sequencing applications require higher cycle counts than the previously mentioned genomic libraries, we next explored whether dye-induced errors might become more pronounced under extended amplification. An *rRNA* gene amplicon control (ZymoBIOMICS Microbial Community DNA Standard) was amplified for 16, 20, 24, or 28 cycles in the presence or absence of SYBR Green I (Thermo Fisher Scientific), and single-base and indel error rates were quantified (Figure 2A). Observed error rates rose markedly with increasing cycle number across all substitution and indel classes, with the highest rates seen at 28 cycles (Figure 2B,C). Against this backdrop, the contribution of SYBR Green I (Thermo Fisher Scientific) was detectable but small, with dye-containing reactions at matched cycle counts showing only slightly elevated error rates relative to the no-dye control. The reduction in cycle-driven error afforded by the reduced cycle counts of the iconPCR instrument (n6tec) should therefore more than offset the small additional error associated with the presence of dye.

We next investigated changes in mutational signatures caused by the presence of dyes in the amplification reaction. Mutational signatures are more sensitive than SNP analysis in that they include the context of the nucleotide change and are frequently highly correlated with the mechanism of action.^7^ Thus, a mutation that is associated with base intercalation would be expected to have a specific signature which could help separate it from the background. Since all observed variants are expected to be technical errors in this reference sample design, any signature enriched in dye-containing conditions would be attributable to the dyes themselves.

**Figure 3.**
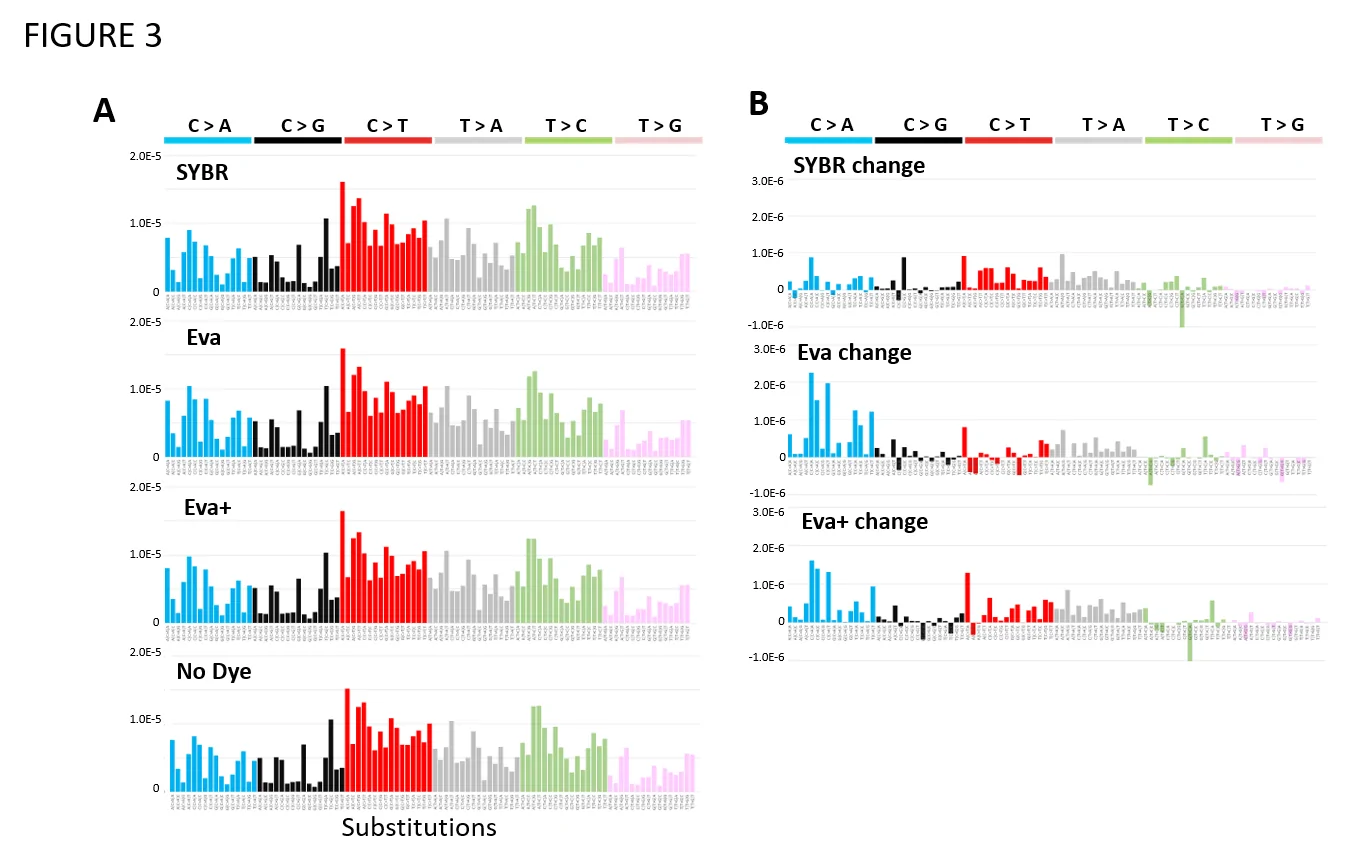
**Single-base substitution spectra and dye-induced changes in libraries amplified with intercalating dyes.** (A) Observed mutational spectra across 96 trinucleotide substitution contexts, normalized to total bases sequenced, for libraries prepared under each dye condition and the no-dye control. Substitution classes (C>A, C>G, C>T, T>A, T>C, T>G) are color-coded as indicated. SYBR: SYBR Green I; Eva: EvaGreen; Eva+: EvaGreen Plus. (B) Dye-imparted substitution changes, calculated by subtracting the no-dye spectrum from each dye-containing condition.

Mutational spectra across all dye conditions were superficially indistinguishable from the no-dye control (Figure 3A). Attempted decomposition of these spectra into known SBS (single-base substitution) signatures using SigProfilerAssignment revealed no dominant signature attributable to dye exposure; since this reaction system is entirely in vitro, a genuine dye-induced mutagen would be expected to produce a simple and recognizable signature. Reconstruction from known SBS types confirmed no systematic difference between conditions. To increase sensitivity, the no-dye spectrum was subtracted from each dye-containing condition to isolate any residual dye-specific signal (Figure 3B). These difference spectra showed only modest increases, predominantly in C>T and T>C transitions across all dyes, with EvaGreen (Biotium) and EvaGreen Plus (Biotium) also showing minor C>A transversions. Attempted signature reconstruction of the difference spectra identified no dominant signal; the highest contributors were SBS4 (tobacco smoke-associated) in EvaGreen (Biotium) (0.54) and SBS8 (unknown etiology) in SYBR (Thermo Fisher Scientific) and EvaGreen Plus (Biotium) (0.486 and 0.421, respectively), with no consistent pattern across conditions. Thus, the differential nucleotide transition signature represents a likely novel artifactual signature.

**Figure 4.**
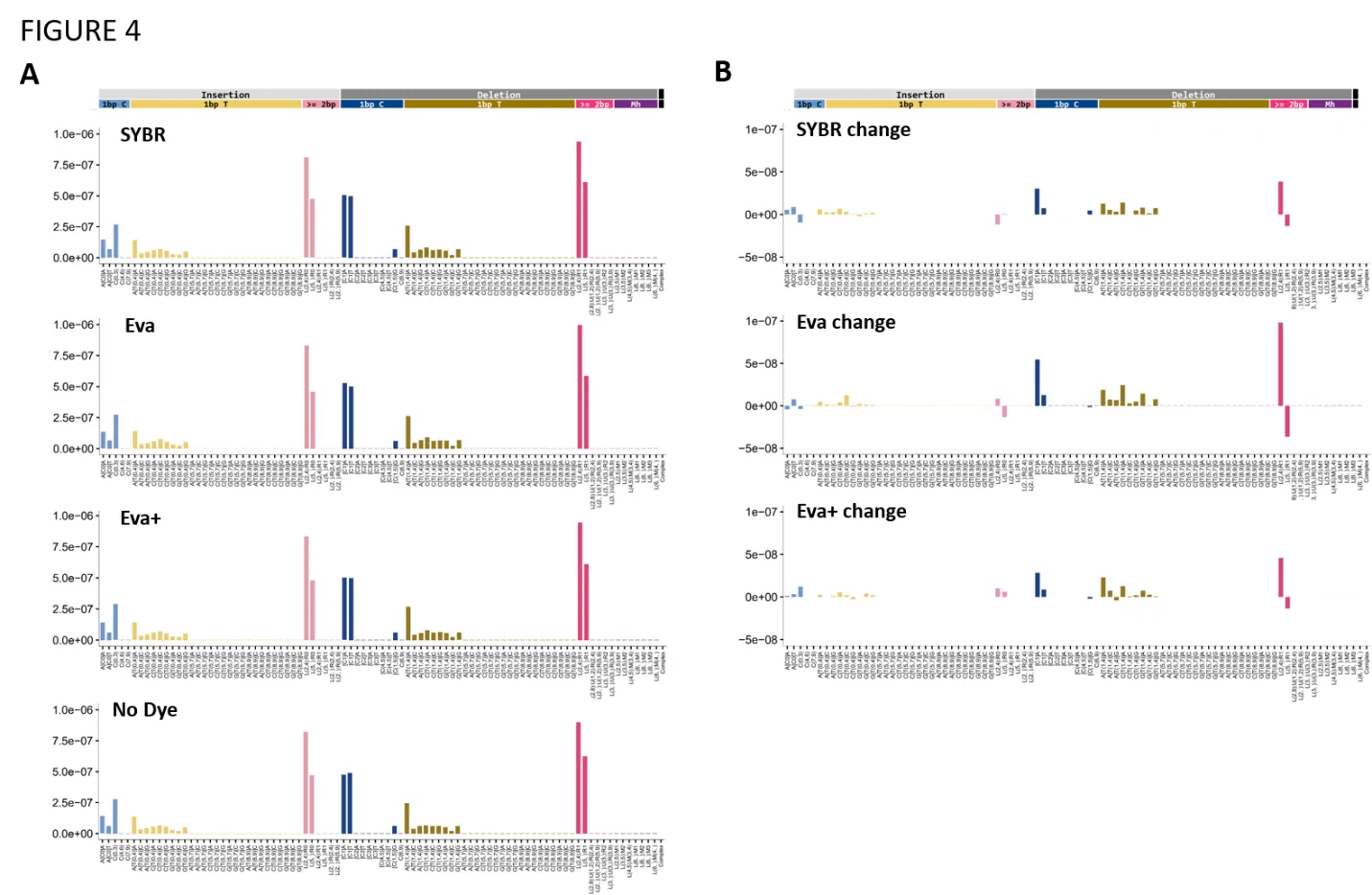
**Indel spectra and dye-induced changes in libraries amplified with intercalating dyes.** (A) Observed indel spectra across insertion and deletion contexts as defined by Koh et al.,^19^ normalized to total bases sequenced, for libraries prepared under each dye condition and the no-dye control. Complex mutations are not shown. SYBR: SYBR Green I; Eva: EvaGreen; Eva+: EvaGreen Plus. (B) Dye-imparted indel changes, calculated by subtracting the no-dye spectrum from each dye-containing condition.

We next looked for changes in indel signatures associated with dye presence in the amplification reaction (Figure 4A). Given that an intercalated dye molecule occupies a position structurally analogous to a base pair, intercalating dyes might be expected to preferentially induce single-base insertions, particularly in repetitive sequence contexts where slippage is most likely. Absolute indel spectra were highly similar across all conditions with no major shift in pattern observed. Consistent with the bulk indel rates observed in Figure 1, deletion events exceeded insertions in all dye conditions; notably, no enrichment of single-base insertion events was observed in any specific sequence context, ruling out a context-specific insertion signal masked by the overall deletion excess. Focusing on the differences between the spectra (Figure 4B) revealed modest but consistent shifts in indel patterns across dye-containing conditions. The most prominent signals were a modest increase in 2–4 bp deletions (~4–10% above the no-dye control, with EvaGreen (Biotium) being the most pronounced) and deletions of a single C preceding an A (~4–10% above the no-dye control). A corresponding decrease in deletions of 5 bp or greater (~2–6% below the no-dye control) was also observed. In all cases, the changes represented a minority of observed mutations.

We next examined whether the observed indel changes corresponded to any known mutational signatures. Reconstruction of the absolute indel spectra identified no dominant signature attributable to dye exposure, with the sum of all assigned signatures reaching a cosine fit of only 0.77 across conditions. An analysis of the difference spectra (Figure 4B), where only dye-induced changes should be represented, yielded even poorer overall fits (0.67–0.71). The largest contributors to these difference spectra included signatures associated with TOP1-linked deletions (InD4a) and APOBEC activity (InD9a), neither of which has a plausible mechanistic link to base intercalation. Attempted modeling against a panel of experimental chemical genotoxin signatures similarly failed to identify a coherent signal, with the highest-scoring match, 6-Nitrochrysene, explaining only a minority of the EvaGreen (Biotium)difference signatures, and cosine fits ranging from 0.45–0.56 across conditions. The indel difference spectrum therefore lacks any coherent signature attributable to dye exposure, consistent with the substitution analysis.

## Discussion

Intercalating dyes present during iconPCR amplification had minimal impact on mutational rates across all analyses performed. In high-fidelity genomic library preparation, single-nucleotide variant and indel rates in dye-containing conditions were close to the background error rate of the no-dye control, with only modest increases in short deletions and no enrichment of single-base insertions, and mutational signature analysis revealed no coherent signature attributable to dye exposure in either substitution or indel space. Extending the analysis to higher cycle counts in a *16S* amplicon system reinforced this conclusion: while observed error rates rose substantially with cycle number, the additional error attributable to the dye remained small at every cycle count tested. Together, these experiments indicate that cycle number, rather than the presence of dye, is the dominant driver of accumulated amplification error. Because these results were obtained under deliberately stringent conditions, with dye concentrations and cycle counts set above the standard iconPCR operating parameters, the rates observed here likely represent an upper bound on real-world dye-induced mutagenicity. One practical advantage of the iconPCR instrument (n6tec) is therefore its reduction in the number of cycles required for many applications, which itself mitigates the formation of artifactual mutations and offsets the very low levels of dye-derived error observed here.

The observed error rates are unlikely to meaningfully impact results in the large majority of NGS workflows, including bulk whole-genome and whole-exome sequencing, RNA-seq, ChIP-seq, and ATAC-seq. Applications that depend on variant detection at the lowest measurable allele frequencies warrant more careful consideration. Minimal residual disease detection requiring discrimination of somatic variants at allele frequencies as low as 0.001–0.01%,^20^ where any systematic elevation in technical error rates could contribute false-positive calls, is one such domain. Single-cell DNA sequencing for clonal analysis, where amplification errors cannot be averaged across a bulk population and are a recognized source of noise,^21^ represents another such application. Finally, de novo mutational signature analysis in translational or mechanistic contexts warrants particular awareness. The consistent pattern of C>T and T>C transitions in the substitution difference spectra, together with the modest enrichment of 2–4 bp deletions in the indel difference spectra, provides a recognizable fingerprint that should make any dye contribution detectable at the level of the mutational spectrum, though it cannot address the origin of any individual variant. Accordingly, caution is warranted in the most sensitive applications operating at the limits of detection, where even a modest shift in the error background may have meaningful consequences for the results.
